# Wheat Biscuits Enriched with Plant-Based Protein Contribute to Weight Loss and Beneficial Metabolic Effects in Subjects with Overweight/Obesity

**DOI:** 10.3390/nu14122516

**Published:** 2022-06-17

**Authors:** Panagiota Binou, Amalia E. Yanni, Klio Kartsioti, Aikaterini Barmpagianni, Panagiotis Konstantopoulos, Vaios T. Karathanos, Alexander Kokkinos

**Affiliations:** 1 Laboratory of Chemistry-Biochemistry-Physical Chemistry of Foods, Department of Nutrition and Dietetics, Harokopio University of Athens, 17671 Athens, Greece; gbinou@hua.gr (P.B.); ds21015@hua.gr (K.K.); vkarath@hua.gr (V.T.K.); 2 1st Department of Propaedeutic Internal Medicine, Laiko General Hospital, Medical School, National and Kapodistrian University of Athens, 11527 Athens, Greece; katerina.barbagianni@gmail.com (A.B.); akokkinos@med.uoa.gr (A.K.); 3 Laboratory of Experimental Surgery and Surgery Research, Medical School, National and Kapodistrian University of Athens, 11527 Athens, Greece; panos2661987@gmail.com

**Keywords:** biscuit, weight loss, plant proteins, branched-chain amino acids, L-arginine, obesity

## Abstract

The present study aimed to assess the impact of daily consumption of a snack fortified with plant proteins with high content in amino acids with appetite regulating properties (BCAAs and L-arginine), as part of a dietary intervention, on weight loss. Seventy adults without diabetes (26 male, 44 female) and with overweight/obesity participated in a 12-week restricted dietary intervention and were randomized to either a control or an intervention group, consuming daily 70 g of conventional wheat biscuits (CB) or an isocaloric amount of wheat biscuits enriched with plant proteins (PB) originating from legumes and seeds, respectively. Anthropometric characteristics were measured and venous blood samples were collected at baseline and at the end of the intervention. Decreases in body weight, body fat mass and waist circumference were observed in both groups. Participants in the intervention group experienced greater weight loss (7.6 ± 2.7 vs. 6.2 ± 2.7%, *p* = 0.025) and marginally significant larger decrease in body fat mass (4.9 ± 2.2 vs. 3.9 ± 2.4 kg, *p* = 0.059). A moderate reduction in IL-1β levels (*p* = 0.081), a significantly higher decrease in TNF-α levels (*p* < 0.001) and a marginally significant greater leptin decrease (*p* = 0.066) in subjects of the PB group were noticed. Greater reductions in caloric and carbohydrate intake and a trend towards a higher decrease in fat intake were also observed in participants of this group. Incorporation of plant-based proteins with high content in amino acids with appetite-regulating properties in wheat biscuits may contribute to greater weight loss and improvement of metabolic parameters in subjects who are overweight or obese. Protein enrichment of snacks offers a beneficial qualitative manipulation that could be successfully incorporated in a diet plan.

## 1. Introduction

The obesity epidemic is one of the major public health challenges; its prevalence is alarmingly rising globally over the last decades despite the increasing knowledge about its health risks and strategies for prevention. According to recent data from the World Health Organization (WHO), the prevalence of worldwide obesity has nearly tripled since 1975. Specifically, almost two billion adults who overweight were recorded, of whom over 650 million had obesity [1].

Adherence to healthy eating patterns can be an effective approach for the management of obesity that focuses not only on weight (and therefore BMI) reduction, but also on waist circumference (WC) reduction and the improvement in body composition by maintaining fat free mass (FFM) and decreasing fat mass [2]. The Mediterranean Diet (MD), a diet dominated by the intake of plant foods (i.e., fruits, vegetables, whole grains, legumes, seeds and unsalted nuts) that are minimally processed, low consumption of red meat and use of olive oil as the principal source of fat [3], appears to be successful in promoting weight loss and is associated with a reduction in all-cause mortality [4].

Legumes, a nutritious staple of the MD, are a valuable food source and have a low energy density. They are an excellent and economical dietary source of high-quality protein, a food with a low glycaemic index (GI), high in dietary fiber (including soluble and insoluble fibers), low in fat and free of saturated fat and cholesterol [5]. Legumes along with seeds are groups of plant protein sources of the MD that constitute promising agents in the development of plant-based products [6]. Consumption of legumes has been associated with an increase in satiety and the management of obesity and its related co-morbidities. Due to the potential health benefits of legumes, scientific interest has risen in recent years, leading to the fortification of foods, especially cereal-based products with legumes, or legume-derived proteins and flours [7].

As mentioned above, legumes and their flours are rich in protein, of which the satiating effect has been proven to be greater than of the other macronutrients, as it increases levels of specific anorectic gut hormones to a greater extent [8,9,10,11]. Therefore, they are rich in amino acids, the structural units of proteins, which have been shown to possess the ability to positively influence metabolic aspects of obesity [12], as they play an important role in the mechanisms involved both in central and peripheral appetite regulation [13,14].

More specifically, studies in human beings have shown positive results following the administration of L-arginine. This amino acid appears to be capable of evoking an anorexigenic response by increasing postprandial responses of the anorectic gut peptides Glucagon-like-peptide-1 (GLP-1) and Peptide tyrosine tyrosine (PYY) in subjects with normal weight or obesity [15,16]. L-arginine supplementation also appears to improve anthropometric parameters (body weight, waist circumference), blood pressure levels and certain blood biochemical indices associated with cardiovascular disease prevention [16,17,18].

Administration of branched-chain amino acids (BCAAs) (i.e., L-leucine, L-isoleucine and L-valine) is also related to suppressed appetite [19]. Notably, enrichment of foods with L-leucine has shown great results regarding appetite perception in human beings, since their consumption leads to increased fullness and reduced hunger [20,21]. L-leucine suppresses food intake through regulation of neuropeptide Y/Agouti related peptide (NPY/AgRP) and proopiomelanocortin (POMC) release [22,23].

In the present study, a wheat biscuit enriched with plant proteins containing high amounts of amino acids with appetite regulating properties (BCAAs and L-arginine) was examined regarding its effects on body weight management and metabolic parameters of subjects who are overweight or obese.

## 2. Materials and Methods

### 2.1. Subjects

The target population of the study included individuals with overweight/obesity (body mass index (BMI) ≥ 25 kg m^−2^), and without diabetes, aged > 18 years, with normal exercise, eating and drinking habits. Exclusion criteria were chronic medical illness (cardiovascular disease, chronic liver, kidney disease, untreated thyroid disease), pregnancy and lactation, use of nutritional supplements that could interfere with the results, a history of drug and/or alcohol abuse or psychiatric disease prohibiting adherence to the protocol.

Seventy subjects participated in the present study and were recruited by means of poster and electronic advertisements. They were enrolled after being informed in detail about its nature and all procedures and giving their written consent for participation. The protocols were reviewed and approved by both the Institutional Review Board/Ethics Committee of Laiko General Hospital and Harokopio University of Athens. The study protocol registration number is ClinicalTrials.gov: NCT04176614.

### 2.2. Study Design

**Figure 1:**
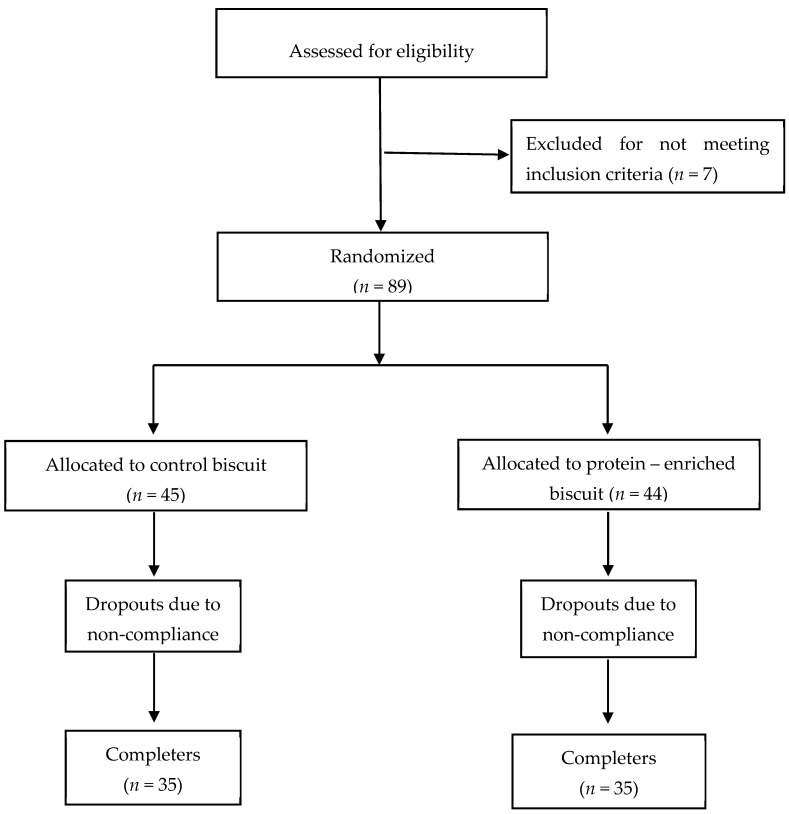
Flow chart of the study.

A dietary intervention was conducted at the 1st Department of Propaedeutic and Internal Medicine, Laiko General Hospital, Medical School, National and Kapodistrian University of Athens, in collaboration with the Laboratory of Chemistry, Biochemistry and Physical Chemistry of Foods, Department of Nutrition and Dietetics, Harokopio University of Athens, Greece. The study was designed as a 12-week, single-blinded, randomized dietary intervention with a total of seventy subjects (*n* = 44 females, *n* = 26 males, Figure 1).

After the run-in period, participants were randomized in two groups to consume either biscuits supplemented with plant proteins originating from plant flours (PB) or a matching placebo [conventional isocaloric wheat biscuits, (CB)]. Volunteers in both groups received dietary counseling sessions and individualized guidance by trained dietitians along with a weekly diet plan. They were asked not to change their exercise habits during the study. A caloric deficit of 20% of their daily energy requirements using an individualized dietary strategy was prescribed. Specifically, the diets were designed to provide the following macronutrient distribution: 45% of energy as carbohydrates, 18% as proteins and 37% as fat. The participants were instructed to follow the prescribed dietary regime for 12 consecutive weeks and were advised to consume 5 biscuits per day (1 biscuit = 14 g), as an afternoon snack (about 3 h after lunch). The biscuits were supplied to the participants at each visit.

Total daily energy intake was calculated based on baseline measurements of weight and resting metabolic rate (RMR), multiplied with a physical activity level (PAL) of 1.4, which indicates a low level of physical activity. An energy intake of 1300–1700 kcal/day was calculated for the female and of 1800–2500 for the male participants. In this context, volunteers received (at the beginning of the intervention weekly and then twice a month) an individualized diet plan with amounts of each food expressed in grams, in both raw and cooked terms, to facilitate comprehension and adherence. Adherence to the dietary intervention was evaluated in each visit by measurement of weight loss and completion of a 3-day weighed dietary record (two weekdays and one weekend day). Participants who did not follow at least 75% of the prescribed diets for two successive weeks were excluded from the study.

Participants completed a series of questionnaires at their baseline screening visit, including demographic information, medical history, body weight history, energy intake [via a 24-h dietary recall and a semi-quantitative food frequency questionnaire (FFQ)] and expenditure data [using the International Physical Activity Questionnaire (IPAQ)]. The 24-h dietary recall, FFQ and IPAQ were also completed at the end of the 12-week study period.

During the first visit, volunteers presented to the laboratory in the morning (between 7.00 and 8.30 a.m.) after an overnight fast. A detailed clinical examination and anthropometric measurements, including body weight, body composition, blood pressure and RMR measurements, were performed at the first and last session. Specifically, body weight was measured with light clothing on an electronic scale (TANITA WB-110MA, Tokyo, Japan) and body composition by bioelectrical impedance analysis (Tanita BC-418, Tokyo, Japan). Height was measured using a stadiometer (Seca Mode 220, Hamburg, Germany) with subjects not wearing shoes, their shoulders in a relaxed position and their arms hanging freely. Waist circumference was determined at the midpoint between the lower margin of the least palpable rib and the top of the iliac crest in a standing position at the end of gentle expiration. Hip circumference measurement was taken around the widest portion of the buttocks. Both waist and hip circumferences were measured in duplicate to the nearest 0.5 cm using a fiberglass tape. RMR was assessed by indirect calorimetry (Fitmate TM Cosmed, Rome, Italy). For the indirect calorimetry assessment, the first five minutes of each test were discarded and the assessment continued until there was a period of 5 consecutive minutes with a coefficient of variation of RMR ≤ 10%.

### 2.3. Blood Analyses

Fasting blood samples were collected at the first and the last session in pre-cooled vacutainers with K_3_EDTA as anticoagulant and centrifuged immediately (3000 rpm for 10 min at 4 °C) for plasma separation. For serum, blood was collected in plain vacutainers, allowed to clot at room temperature for 30 min and then centrifuged (3000 rpm for 10 min at 4 °C). After isolation, plasma and serum were stored at −80 °C until analysis.

Measurement of glycated haemoglobin (HbA_1_c) and basal biochemical measurements including plasma glucose and serum total cholesterol (TC), high-density lipoprotein cholesterol (HDL-C), triacylglycerols (TAG), alanine aminotransferase (ALT), aspartateaminotransferase (AST), γ-glutamyl transferase (γ-GT), urea, creatinine, uric acid and total proteins were performed on an automated biochemical analyzer (Medilyzer, Medicon Hellas, Athens, Greece), using commercially available diagnostic kits. Low-density lipoprotein cholesterol (LDL-C) was calculated using Friedewald’s formula.

In plasma, leptin was assayed by a sandwich ELISA method on a microtiter plate using a commercially available human leptin kit (Human Leptin ELISA kit; Millipore, MA, USA). Adiponectin was similarly detected by a sandwich ELISA method using commercially available kits (Human Adiponectin ELISA kit; Millipore, MA, USA). CRP, TNF-α,IL-6 and IL-1β were also determined by a sandwich ELISA method using commercially available high-sensitivity kits (Human hs C-Reactive Protein/CRP ELISA kit; R&D Systems, Human hsTNF-α ELISA kit; R&D systems, Human hsIL-6 ELISA kit; R&D systems, Human hs IL-1β ELISA kit; R&D systems, Minneapolis, MN, USA, respectively).

### 2.4. Test Biscuits

Two different wheat biscuits were developed. The PB was a cocoa-flavored biscuit enriched with plant proteins. It was formulated by substituting 30% of white wheat flour with plant flours originating from legumes and seeds used in the MD. The CB was a conventional cocoa-flavored biscuit prepared using white wheat flour and served as control. The biscuits were isocaloric and contained a similar amount of fat. Compared to the CB, the PB had a higher content of protein (and individual amino acids) and fiber derived from plant flours. It was approximately 50% richer in total amino acids, and regarding the amino acids that have been associated with appetite regulation, it was about 50% richer in BCAAs and 75% richer in L-arginine.

The nutritional composition of the two tested biscuits is presented on Table 1. Nitrogen (protein: Nx6.25) was measured by Kjeldahl (ISO 1871) and fat by Soxhlet procedures. Total dietary fibers were determined by the AOAC method991.43. Amino acids were separated by ion exchange chromatography and determined by reaction with ninhydrin using photometric detection (EU 152/2009).

### 2.5. Statistical Analysis

It was estimated via power analysis calculations that a sample of a total of 70 subjects would allow for the detection of a 2 kg difference between groups, with a power of 80%. By assuming a 20% drop-out rate, 88 volunteers were enrolled and 70 completed the 12-week intervention.

The Kolmogorov–Smirnov test was used to check for normal distribution of the data. Descriptive statistics are presented as mean ± SD and the results as mean ± SEM. A paired sample *t*-test was used to compare anthropometric and biochemical parameters at the beginning and the end of the intervention in each group. Regarding differences between the two groups, an independent samples *t*-test was performed. The correlations between anthropometric characteristics, biochemical indices, hormone levels and inflammatory markers were statistically evaluated using Pearson correlation (PC) tests. *p* < 0.05 was considered statistically significant. The SPSS 21.0 statistical software package (IBM, New York, NY, USA) was used for analyses.

## 3. Results

### Study Results

The baseline anthropometric, clinical and biochemical characteristics of the subjects of the two groups who successfully completed the intervention are presented in Table 2. The mean age of subjects in both groups was about 45 years old, with a mean BMI a little over 30 kg/m^2^ and a high percentage of body fat. There were no significant differences in any of the baseline characteristics between participants of the two groups.

Anthropometric and biochemical measures of the two groups at the end of the intervention are also outlined in Table 2. Statistically significant decreases in body weight (and therefore BMI), waist circumference and body fat were reported in both groups (*p* < 0.001). In addition, a modest but significant reduction in lean body mass was observed in both groups. Subjects also displayed significant reductions in arterial blood pressure, whereas there was a significant decrease in AST and ALT values (*p* = 0.018 and *p* = 0.044, respectively) in participants allocated in the group of the plant protein-enriched biscuit. No between-group differences were identified for the biochemical indices.

Table 3 presents comparisons in anthropometric characteristic changes between groups at 12 weeks. Participants in the PB group experienced a significantly greater decrease in body weight and BMI (*p* = 0.025 and *p* = 0.038, respectively) compared to the participants in the CB group. Moreover, a trend towards a greater reduction in body fat mass for subjects in the PB group (*p* = 0.059) was observed, whereas no statistically significant difference regarding waist circumference decrease between groups was reported.

Regarding caloric and macronutrient intake (Table 4), there were no significant differences in baseline values between the two groups and decreases were observed in both. However, greater reductions in caloric and carbohydrate intake (*p* = 0.009 and 0.018) and a trend towards a higher decrease in fat intake (*p* = 0.078) were reported in participants of the PB group.

Changes in levels of hormones and inflammatory markers are presented in Table 5. Leptin levels were significantly reduced in both groups (*p* < 0.001), while TNF-α levels were only reduced in the PB group (*p* < 0.001). In addition, a slight but significant decrease in adiponectin levels was reported in participants of the CB group. While there were no significant changes over time in hs-CRP and IL-6 levels, a moderate decrease in IL-1β levels in participants of the PB group was observed (*p* = 0.081). A significantly higher TNF-α level decrease (*p* < 0.001) and a trend towards a greater leptin decrease (*p* = 0.066) in subjects of the PB group were noted.

Regarding correlations between parameters examined in the present study, a few associations in both control and intervention groups were observed. Weight loss was significantly related to BMI decrease, waist circumference decrease and fat mass decrease (Control Group: PC = 0.959, PC = 0.574, PC = 0.802, respectively, *p* < 0.001; Intervention Group: PC = 0.930, PC = 0.622, PC = 0.725, respectively, *p* < 0.001). Moreover, the reduction in CRP levels was correlated with the reduction in waist circumference in the intervention group (PC = 0.460 *p* = 0.005).

## 4. Discussion

The rapidly increasing rate of obesity and its related co-morbidities over the last years have created an urgent need for finding strategies to improve prevention and treatment. The development of products rich in proteins, which increase the levels of specific anorectic peptides to a greater extent than other macronutrients, thus leading to better appetite regulation [12,13,15,23], arises as a potential useful practice. Legumes and seeds, the consumption of which has been associated with potential health benefits [5,6,7], are rich in protein and therefore their use in the development of new products seems to have promising prospects.

In the present study, a dietary intervention examining two types of biscuits resulted in significant weight loss over a 12-week period in both groups as a reduction in energy intake was achieved. All subjects, regardless of the group they were enrolled into, experienced beneficial changes in waist circumference, body fat mass and arterial blood pressure. However, in the study group, where the enriched biscuit was used as a snack, better results were reported. More specifically, the participants of this group had a reduction in body weight of about 7.6% compared to 6.2% in the control group and there was a trend for greater body fat mass decrease in comparison to the participants of the control group.

The greater reduction in body weight of the subjects in the intervention group occurred because of the significantly higher decrease in caloric intake. This may possibly be due to better appetite regulation offered by the enriched biscuit they consumed as a snack. The aforementioned biscuit was rich in protein and contained approximately twice the amount of protein compared to the control wheat biscuit (9.7 g and 5.1 g per daily snacking portion of 70 g-, respectively). It also contained a higher amount of dietary fiber; however, the difference per serving (2.1 g per 70 g) was not significant enough to explain the superior results achieved by the subjects in the intervention group.

The observed effects are attributed to the dietary intervention with PB and not to an effect of physical activity since as it is shown from the analysis of the IPAQ, the physical activity was not significantly modified during the 12 weeks and did not differ significantly between the two groups. It remained at low levels throughout the intervention. It was also considered that both groups should receive dietary counseling sessions and individualized guidance by trained dietitians along with a weekly diet plan, which was designed to provide the same macronutrient distribution.

Previous studies have examined the impact of differences in the macronutrient composition of snacks on appetite and energy intake. Leidy et al. showed that consumption of high protein afternoon snacks improves appetite control, leading to reduction of high fat evening snacking compared to the consumption of snacks with lower protein content [24]. Similar results were found in two studies that suggested that a small high protein afternoon snack might delay or reduce the portion of subsequent snacking and prevent over-eating later in the day [25,26]. The positive effects of high protein snacks on appetite control compared to conventional high fat or high carbohydrate snacks have also been underlined by other recent studies in volunteers who are overweight or obese [27,28]. Therefore, regarding the results of the present study, taking into consideration that both groups received the same treatment, except for the type of isocaloric biscuit snacks that differed in their macronutrient composition and specifically their protein content, we could assume that the high protein biscuit improved appetite control and led to lower caloric, carbohydrate and fat consumption throughout the day.

More specifically, regarding the amino acids that have been associated with appetite regulation, the enriched biscuit was rich in L-arginine and BCAAs. Administration of BCAAs is related to suppressed appetite. L-leucine is particularly effective at stimulating mTOR signaling [29]; thus, it is possible that this signaling effect underlies its impact on food intake.

L-arginine has been shown to have a delayed and sustained anorectic effect in animals. Particularly, stimulation with 10 mM of L-arginine significantly increases GLP-1, PYY and GIP secretion in isolated rat small intestine tissue [30]. Alamshah et al. have reported a reduction in food intake when food was offered 8 h after administration of L-arginine in rodents [31]. In another study L-arginine was shown to stimulate CCK and GIP secretion from porcine intestinal tissue [32].

Regarding human studies, it has been shown that there may be delayed effects of L-arginine on appetite. In a recent study, ingestion of 3 g of L-arginine in combination with an ad libitum meal (consumed 60 min post L-arginine ingestion) led to significant elevation of GLP-1 and PYY in healthy human volunteers compared to the control group [15]. L-arginine seems to increase postprandial release of anorectic gut hormones as it is possibly sensed by amino-acid-sensing receptors in the gut, which then intercede its effect on gut hormone release. Some of these receptors are expressed on the same enteroendocrine cells that express GLP-1 and PYY [33,34].

In the present study, changes in levels of obesity-related hormones and inflammatory markers were also measured. Leptin levels were significantly reduced in both groups. Our findings are in accordance with the existing literature, as it has been shown that weight loss is related with reduction in leptin concentrations [35,36,37]. In obesity, despite increased leptin concentrations, the efficacy of the anorexic effect of this adipokine is decreased [38,39] and leptin resistance is developed due to a defect in intracellular signaling associated with the leptin receptor or decreases in leptin transport across the blood–brain barrier [40]. Fasting concentrations of adiposity signals such as leptin, which are increased with adiposity, are subsequently reduced during caloric restriction and weight loss [41]. Weight loss has been found to modify the average leptin synthesis level [42]. Specifically, a person in the process of weight loss will reduce their body fat percentage and down-regulate leptin levels to promote hypothalamus response sensitivity [37].

Regarding inflammatory markers, which were studied since obesity is linked with a chronic low-grade inflammatory state, a moderate decrease in IL-1β levels and a significant decrease in TNF-α levels in participants of the PB group over time were observed. Moreover, significant differences in the TNF-α reduction levels and moderate differences regarding leptin reduction levels between the two groups were reported at the end of the intervention. In both occasions, the decrease was greater for the participants of the PB group. These findings seem to be explained by the greater weight loss experienced by subjects in this group, since weight reduction has proven to be beneficial to the reduction of low-grade inflammation [43,44]. Due to caloric restriction, changes on gene expression of cytokines are caused, leading to the reduction of IL-1b expression and the improvement of insulin sensitivity [45], and gene expression in adipose tissue is regulated, resulting to an increase in mitochondrial function [46]. Pro-inflammatory genes are down-regulated after weight loss and the expression of cytokines is inhibited [47]. The greatest improvements were observed in studies where a weight loss of at least 10% was achieved [44].

Except for the weight loss, this difference may also be attributed to some extent to the higher BCAAs and L-arginine content of the enriched snack, which was consumed by the participants daily. BCAAs have been shown to play an important role in the anti-inflammatory process. More specifically, supplementation of leucine and pyridoxine in individuals with obesity led to an increase in the anti-inflammatory adipokine adiponectin [48]. Ohno et al. have reported a decrease in CRP levels in patients with liver cirrhosis after oral administration of BCAAs granules [49]. Regarding L-arginine, studies in animal models have reported a significant decrease in IL-1β and/or TNF-α levels in the groups that received treatment with L-arginine in comparison to the untreated groups [50,51]. Another study in human subjects has shown a negative association between L-arginine consumption and CRP levels [52]. The anti-inflammatory action of L-arginine could be attributed to its enhancing effect on nitric oxide production that was reported to have anti-inflammatory properties through gene expression regulation and cell cytokine inhibition in the vascular system [53]. However, it should be noted that in the aforementioned human studies the amino acid supplementation was in greater amounts compared to our study.

With respect to the associations reported in our study between weight loss and reduction of weight circumference and body fat mass, they have been consistent with the previous literature. Except for weight loss being closely related to waist circumference reduction, adherence to the MD has been associated with beneficial effects on body weight and waist circumference [54,55,56,57]. Moreover, in the intervention group, the reduction of CRP has been associated with decreased waist circumference. In a previous study, waist circumference was shown to be the variable related to metabolic syndrome with the strongest correlation with CRP [58]. Other studies have also reported associations between systemic inflammation and greater adiposity as measured by waist circumference and BMI [59,60]. A limitation of the present study is that the physical activity levels and dietary habits were assessed via IPAQ and FFQ questionnaires, respectively, which are self-reported methodologies with well-known disadvantages. Moreover, this was a short-term dietary intervention. A longer intervention with a follow-up period could help to evaluate the potential efficacy of the PB more clearly in body weight management. In conclusion, wheat biscuits enriched with plant proteins with high amounts of amino acids with appetite-regulating properties constitute a snack alternative that could contribute to a greater adherence to a hypocaloric diet plan. Consumption of functional biscuits by subjects who are overweight or obese results in higher weight reduction accompanied by improved metabolic effects.

The positive results of high protein diets compared to low protein diets are well documented [61,62,63], and protein enrichment of popular snacks offers a more qualitative snacking approach that could be successfully incorporated in a diet plan and also be addressed to a wider consumer group.

## Figures and Tables

**Table 1:** Nutritional composition of the two biscuits.

Biscuit	Energy Content (Kcal)	Carbohydrates (g)	Total Dietary Fiber (g)	Protein (g)	BCAAs (g)	Arg (g)	Fat (g)	Saturated Fatty Acids (g)	Monounsaturated Fatty Acids (g)	Polyunsaturated Fatty Acids (g)
			*per 100 g*							
CB	445	71.0	1.5	7.3	0.98	0.24	14.0	6.70	5.19	2.11
PB	451	56.8	5.6	14.5	2.05	1.05	17.2	7.70	6.70	2.80

Data are presented per 100 g. CB, control wheat biscuit, PB wheat biscuit enriched with plant proteins, BCAAs Branched-Chain Amino Acids, Arg Arginine.

**Table 2:** Anthropometric, clinical and biochemical characteristics of the subjects at baseline and after 12 weeks.

	Control Biscuit			Enriched Biscuit		
Characteristic	Baseline	Endpoint	p-Value *	Baseline	Endpoint	p-Value *
Sex (male/female)	14/21	14/21		12/23	12/23	
Age (years)	46.0 ± 9.1	46.0 ± 9.1		42.9 ± 14.7	42.9 ± 14.7	
Weight (kg)	85.1 ± 13.3	79.9 ± 12.5	**<0.001**	85.3 ± 16.7	78.8 ± 15.4	**<0.001**
BMI (kg/m^2^)	30.9 ± 3.7	29.0 ± 3.7	**<0.001**	30.6 ± 4.2	28.2 ± 3.7	**<0.001**
WC (cm)	99.6 ± 13.6	93.7 ± 13.0	**<0.001**	97.0 ± 13.2	90.2 ± 12.4	**<0.001**
Body fat (%)	35.1 ± 8.1	32.5 ± 9.4	**<0.001**	36.1 ± 6.4	32.7 ± 6.5	**<0.001**
Body fat mass (kg)	29.9 ± 8.5	26.0 ± 9.2	**<0.001**	30.9 ± 8.1	25.8 ± 7.3	**<0.001**
Lean mass (kg)	55.3 ± 11.4	53.8 ± 11.2	**<0.001**	54.8 ± 12.8	53.3 ± 12.2	**<0.001**
Physical activity (min/week)	137.3 ± 104.7	125.1 ± 105.3	0.156	145.6 ± 88.0	146.9 ± 84.9	0.895
SBP (mmHg)	120.3 ± 12.2	113.3 ± 11.5	**0.001**	118.8 ± 18.1	108.8 ± 22.0	**0.027**
DBP (mmHg)	81.0 ± 9.9	77.8 ± 9.0	**0.029**	79.9 ± 10.6	74.7 ± 8.2	**<0.001**
Glucose (mg/dL)	90.4 ± 12.6	90.3 ± 6.3	0.989	90.6 ± 11.9	90.1 ± 10.9	0.677
Cholesterol (mg/dL)	185.0 ± 35.6	189.6 ± 38.5	0.364	179.8 ± 32.9	181.1 ± 32.4	0.732
HDL-C (mg/dL)	59.7 ± 9.6	59.5 ± 9.2	0.174	59.8 ± 10.9	60.0 ± 10.4	0.386
LDL-C (mg/dL)	104.6 ± 34.7	108.9 ± 38.2	0.354	102.3 ± 30.5	103.6 ± 28.9	0.707
Triacylglycerols (mg/dL)	103.6 ± 63.6	99.3 ± 41.3	0.595	87.4 ± 43.2	89.1 ± 44.9	0.746
AST (U/L)	17.0 ± 5.6	16.9 ± 4.2	0.888	19.7 ± 11.1	15.8 ± 4.8	**0.018**
ALT (U/L)	16.7 ± 8.9	17.7 ± 5.5	0.417	18.9 ± 14.1	15.0 ± 6.2	**0.044**
γ-GT (U/L)	23.8 ± 13.8	24.5 ± 12.8	0.611	21.7 ± 9.9	20.1 ± 9.5	0.135
Urea (mg/dL)	30.2 ± 7.5	29.1 ± 5.9	0.382	29.7 ± 5.0	30.5 ± 5.8	0.444
Creatinine (mg/dL)	1.0 ± 0.1	1.0 ± 0.1	0.697	1.0 ± 0.1	0.9 ± 0.1	0.400
Uric acid (mg/dL)	4.3 ± 1.1	4.5 ± 1.1	0.378	4.5 ± 1.5	4.5 ± 1.4	0.800
Total proteins (mg/dL)	7.0 ± 0.3	7.0 ± 0.2	0.821	6.9 ± 0.4	6.9 ± 0.3	0.903

Values are expressed as mean ± SD. * Refers to *p-value* < 0.05. *p-value* was calculated via paired samples *t*-test. BMI body mass index, WC waist circumference, SBP systolic blood pressure, DBP diastolic blood pressure, HDL-C high-density lipoprotein cholesterol, LDL-C low-density lipoprotein cholesterol, AST aspartate aminotransferase, ALT alanine aminotransferase, γ-GT γ-glutamyl transferase.

**Table 3:** 12-week changes in anthropometric characteristics between the subjects of the two groups.

Characteristic	Control Biscuit	Enriched Biscuit	p-Value *
Weight loss (%)	6.2 ± 2.7	7.6 ± 2.7	**0.025**
BMI decrease (kg/m^2^)	1.9 ± 0.9	2.4 ± 0.9	**0.038**
WC decrease (cm)	5.9 ± 3.4	6.8 ± 3.1	0.262
Body fat percentage decrease (%)	2.6 ± 2.3	3.3 ± 2.1	0.181
Body fat mass decrease (kg)	3.9 ± 2.4	4.9 ± 2.2	0.059

Values are expressed as mean ± SD. * Refers to *p-value* < 0.05. *p-value* was calculated via independent samples *t*-test. BMI body mass index, WC waist circumference.

**Table 4:** Energy and nutrient consumption and energy expenditure of the subjects at baseline and at the end of the intervention.

	Baseline			Endpoint		
Characteristic	Control Biscuit	Enriched Biscuit	p-Value *	Control Biscuit	Enriched Biscuit	p-Value *
Calorie intake (kcal)	2529.2 ± 471.1	2463.3 ± 441.1	0.554	1917.3 ± 262.3	1736.2 ± 291.6	**0.009**
Protein intake (g)	91.6 ± 22.5	87.2 ± 19.2	0.390	80.1 ± 14.7	76.8 ± 14.0	0.347
Carbohydrate intake (g)	239.8 ± 56.8	226.4 ± 57.6	0.336	210.8 ± 41.8	186.4 ± 41.2	**0.018**
Fat intake (g)	134.0 ± 22.9	134.4 ± 22.9	0.940	82.5 ± 10.1	77.9 ± 11.0	0.078
Physical activity (min/week)	137.3 ± 104.7	145.6 ± 88.0	0.721	125.1 ± 105.3	146.9 ± 84.9	0.345

Values are expressed as mean ± SD. * Refers to *p-value* < 0.05. *p-value* was calculated via independent samples *t*-test.

**Table 5:** Changes in levels of hormones and inflammatory markers for the two groups.

	Control Biscuit			Enriched Biscuit			
Characteristic	Baseline	Endpoint	p-Value *	Baseline	Endpoint	p-Value *	p-Value * (Endpoint between Groups)
Adiponectin (mg/L)	10.8 ± 0.8	9.9 ± 0.8	**0.013**	10.8 ± 1.0	10.7 ± 1.3	0.938	0.598
Leptin (ng/mL)	43.9 ± 5.0	31.1 ± 27.6	**<0.001**	45.9 ± 4.8	26.0 ± 2.7	**<0.001**	0.347
hs-CRP (mg/L)	2.2 ± 0.5	2.5 ± 0.6	0.366	2.3 ± 0.5	2.0 ± 0.3	0.332	0.405
IL-6 (pg/mL)	2.4 ± 0.2	2.8 ± 0.4	0.166	2.4 ± 0.2	2.2 ± 0.1	0.443	0.139
IL-1β (pg/mL)	0.3 ± 0.1	0.2 ± 0.0	0.276	0.7 ± 0.2	0.3 ± 0.1	0.081	0.470
TNF-a (pg/mL)	0.4 ± 0.0	0.4 ± 0.0	0.953	0.5 ± 0.0	0.3 ± 0.0	**<0.001**	0.394
Adiponectin decrease (mg/L)		−0.9 ± 0.4			−0.1 ± 0.6		0.233
Leptin decrease (ng/mL)		−12.8 ± 2.2			−19.9 ± 3.1		0.066
hs-CRP decrease (mg/L)		+0.4 ± 0.4			−0.3 ± 0.3		0.189
IL-6 decrease (pg/mL)		+0.4 ± 0.3			−0.2 ± 0.2		0.107
IL-1β decrease (pg/mL)		−0.1 ± 0.1			−0.4 ± 0.2		0.170
TNF-a decrease (pg/mL)		0.0 ± 0.0			−0.2 ± 0.1		**<0.001**

Values are expressed as mean ± SD. * Refers to *p-value* < 0.05. *p-value* was calculated via paired samples *t*-test and *p-value* (Endpoint between groups) via independent samples *t*-test.

## Data Availability

Data available on request from the authors.
